# Documented caries treatment and prevention among highly caries-active adults: a retrospective observational study of public dental records

**DOI:** 10.1186/s12903-026-09170-0

**Published:** 2026-07-08

**Authors:** Sara Björns, Olga Jensen

**Affiliations:** 1 Department of Preventive and Community Dentistry, Public Dental Service, Region Västra, Gothenburg, Sweden; 2 Department of Cariology, Institute of Odontology, Sahlgrenska Academy, University of Gothenburg, Gothenburg, 405 30 Sweden; 3 Department of Research and Development, Public Dental Service, Region Västra, Gothenburg, Sweden

**Keywords:** Caries epidemiology, Highly caries-active adults, Caries prevention, Caries treatment, Dental records, Quality of care

## Abstract

**Background:**

Evidence-based caries management emphasises identification of disease drivers and causal, preventive, and behaviour-oriented care. However, limited evidence is available on how such care is documented for adults with high caries activity in routine public dental care. This study examined documentation of caries treatment and preventive measures consistent with evidence-based caries management, and whether documented care addressed underlying causes of caries disease.

**Methods:**

This retrospective observational record-review study included 418 adults aged 25–65 years from 19 Public Dental Service clinics in Region Västra Götaland, Sweden. High caries activity was defined as ≥ 4 manifest caries lesions at a complete oral examination in 2015 or 2016. Documented caries-related treatment, preventive measures, causal investigations, and counselling content were extracted from electronic dental records between the index examination and the subsequent complete oral examination. Descriptive statistics summarised documentation patterns. Sex-related differences were analysed using Fisher’s exact test and Mann–Whitney U-test, and associations with age and sex were explored using logistic regression.

**Results:**

The sample comprised 185 women (44.2%) and 233 men (55.7%). Overall, 330 patients (78.9%) had at least one documented preventive and/or treatment measure, whereas 88 patients (21.1%) had none. Oral hygiene advice (43.5%; 95% CI 38.7–48.4) and fluoride advice (40.9%; 95% CI 36.2–45.8) were the most frequently documented preventive measures. Dietary enquiry was documented more often than dietary advice (34.4% vs. 16.3%). Professionally applied fluoride treatment was documented for 15.6%. No record contained documentation indicating a theory-based behaviour change approach or salivary secretion measurement. In exploratory analyses, no statistically significant sex-related difference was observed, whereas increasing age was associated with a higher likelihood of documentation.

**Conclusions:**

Among highly caries-active adults in routine public dental care, documentation showed partial alignment with guideline-relevant preventive domains but limited evidence of causal, intensified, and behaviour-oriented caries management. The findings indicate a documentation and implementation gap in adult caries care and provide a baseline for evaluating guideline-aligned preventive practice.

**Supplementary Information:**

The online version contains supplementary material available at 10.1186/s12903-026-09170-0.

## Background

Untreated dental caries in permanent teeth remains one of the most prevalent oral conditions globally [1]. Dental caries is a multifactorial, biofilm-mediated, sugar-driven, non-communicable disease in which biological, behavioural, social, and environmental factors interact to determine disease development and progression [2–7]. Internal aggregate data from the Public Dental Service in Region Västra Götaland showed that approximately 146,000 examined individuals were registered with manifest caries in 2015 and 2016, corresponding to approximately 23% of examined patients [8]. Preventive caries management includes primary prevention of lesion development and non-invasive or minimally invasive management aimed at arresting or slowing lesion progression [9, 10]. Contemporary caries management requires systematic identification of modifiable disease drivers, including sugar intake, oral hygiene, fluoride exposure, salivary factors, and behavioural and social conditions, followed by preventive measures tailored to the individual risk profile [2–4, 7, 10, 11].

For patients with active caries or high caries risk, Swedish evidence assessments and national guidelines support causal and prevention-oriented caries management, including risk assessment, fluoride-based prevention, dietary measures, and behaviour-oriented counselling [10, 12, 13]. Causal caries management aims to identify biological, behavioural, and environmental factors that contribute to disease development and progression, including frequent exposure to fermentable carbohydrates, biofilm accumulation, fluoride exposure, and modifying host factors such as reduced salivary secretion, in order to establish an individual risk profile and guide tailored preventive management [2, 3, 10–13]. According to the Swedish National Board of Health and Welfare, only 6.4% of patients who received restorative caries care were reported to have received treatment and/or measures to prevent future caries [14]. Earlier studies have shown that dentists’ treatment philosophies, use of caries-risk information, and management of routine oral examinations may vary in ways that affect preventive decision-making [15–17]. Swedish qualitative work has also shown that oral health professionals may assume that patients already understand how to use fluoride toothpaste effectively [18]. Swedish record-review evidence from child and adolescent dental care has shown incomplete documentation of preventive information, including oral hygiene and dietary habits [19].

The Swedish National Guidelines for adult dental care in 2011 emphasised causal and prevention-oriented care for patients with caries risk or active caries; the updated 2022 guidelines retained this prevention-oriented logic [12, 13]. Key recommendations include twice-daily toothbrushing with fluoride toothpaste, reduced sugar intake, professionally applied fluoride treatment, including fluoride varnish, and advisory counselling or theory-based behaviour change approaches rather than oral health information alone [12, 13].

Evaluating routine documentation of caries management is important because dental records provide information on whether preventive and treatment-related care is made visible, traceable, and available for continuity and quality assessment. However, documentation should not be interpreted as a direct measure of all care delivered; rather, it reflects what was recorded in routine clinical practice. To our knowledge, no previous peer-reviewed study has examined the documented delivery of evidence-based caries treatment and prevention among highly caries-active adults in Swedish public dental care using regional electronic dental records.

The primary objective of this study was to examine the documentation of caries treatment and preventive measures consistent with evidence-based caries management among adults with high caries activity in routine public dental care. A second objective was to describe whether documented care addressed modifiable, guideline-relevant drivers of caries disease, including oral hygiene, fluoride exposure, diet, salivary factors, and behaviour-oriented support. A secondary exploratory objective was to assess whether documentation of preventive and/or treatment measures differed by sex or age.

For the exploratory comparative analyses, the null hypotheses were that documentation of preventive and/or treatment measures would not differ between women and men, and that documentation of preventive and/or treatment measures would not be associated with age. Because assessment of causal documentation domains was descriptive, this part of the study was not formulated as a statistical hypothesis.

## Methods

### Study design

This was a retrospective observational record-review study based on routinely collected electronic dental records. The study is reported in accordance with the Strengthening the Reporting of Observational Studies in Epidemiology (STROBE) statement [20] and the REporting of studies Conducted using Observational Routinely collected health Data (RECORD) guidelines [21]. No large language models or artificial intelligence tools were used in the design, analysis, or writing of this manuscript.

### Study setting

The study was conducted within the Public Dental Service in Region Västra Götaland, Sweden. Region Västra Götaland is a large Swedish region with approximately 1.7 million inhabitants and publicly organised dental clinics across urban and rural municipalities. During the study period, the Public Dental Service provided general adult dental care in the region. Nineteen Public Dental Service clinics were included to capture variation in geographic location and socioeconomic context. Nine clinics were located in Gothenburg, and ten were located in smaller cities and communities.

### Study population

Eligible records were identified among adults aged 25–65 years who were regular attenders in the Public Dental Service, paid for their own dental care, and had high caries activity documented at a complete dental examination in 2015 or 2016. High caries activity was defined as ≥ 4 manifest caries lesions. The first eligible complete examination during the inclusion period was defined as Exam 1, and the subsequent complete examination was defined as Exam 2. The observation interval comprised the period between Exam 1 and Exam 2.

A regular dental attender was defined as an individual with more than two complete dental examinations at the same clinic. Records were excluded if the patient was younger than 25 years, older than 65 years, did not pay for adult dental care under the standard self-payment model, did not meet the definition of regular dental attender, or lacked a subsequent complete dental examination. After application of the eligibility criteria, 418 electronic dental records were included in the final analytic sample.

### Data source and extraction

Data were extracted from the regional electronic dental record system T4 (Carestream Dental AB; Stockholm, Sweden), used by the Public Dental Service in Region Västra Götaland during the study period. The T4 record includes administrative, financial, dental-care, and attachment modules; the dental-care module contains structured sections for status/diagnosis, treatment, risk assessment, and daily clinical notes. In the present study, data were extracted from structured dental record fields, procedure codes, and daily clinical notes documented between Exam 1 and Exam 2. The extraction was performed by an information technology specialist employed within the regional organisation. Before transfer to the research dataset, patient- and provider-identifying information was removed in accordance with the ethics approval and applicable data protection regulations.

For each eligible record, data were extracted on age at Exam 1, recorded sex, clinic, regular-attender status, payment model, dental status, caries-related diagnoses, procedure codes for caries treatment and preventive measures, risk assessment information where available, and clinical notes documented between Exam 1 and Exam 2. Manifest caries was defined as a lesion extending into dentine and requiring operative treatment, based on clinical and/or radiographic documentation. Caries-related treatment and preventive measures were identified using predefined procedure codes and corresponding daily clinical notes (Table 1). The documentation domains were defined a priori based on guideline-relevant components of caries management: oral hygiene, fluoride exposure, dietary factors, salivary factors, professionally applied fluoride treatment, supplementary fluoride self-care recommendations, microbiological testing, and behaviour-oriented counselling or treatment.

The record review was performed by one licensed dental professional. To enhance data validity, extracted procedure codes were cross-checked against the corresponding clinical notes. Any discrepancies between procedure codes and narrative documentation were resolved through review of the full record entry.

### Statistical analysis

A sample-size calculation was performed to estimate the proportion of records with documented caries-related treatment and/or preventive measures with acceptable precision. The calculation was based on a finite source population of 2,988 adults in Region Västra Götaland with ≥ 4 manifest caries lesions in 2015. Assuming a 95% confidence level, a 5% margin of error, and a conservative expected proportion of 50%, the required sample size was 341 records after finite population correction. A total of 418 electronic dental records were extracted and reviewed, exceeding the minimum required sample. Analyses by sex and age were exploratory and were not powered to establish equivalence between women and men.

Descriptive statistics were used to summarise patient characteristics and documentation of caries-related treatment and preventive measures. Categorical variables are presented as frequencies and percentages, with 95% confidence intervals where relevant. Continuous variables are presented as mean and standard deviation or median and interquartile range, depending on distribution.

Differences between women and men were analysed using Fisher’s exact test for dichotomous variables and the Mann–Whitney U-test for continuous or ordinal variables. Associations between age and documentation of preventive and/or treatment measures were analysed using age as a continuous variable where appropriate. Binary logistic regression was used to assess the association between recorded sex, age, and documentation of at least one preventive and/or treatment measure. An interaction term between recorded sex and age was included to assess whether the association between age and documentation differed between women and men.

Exact 95% confidence intervals for proportions were calculated using the Clopper–Pearson method. Two-sided tests were used, and p-values < 0.05 were considered statistically significant. P-values are reported to three decimal places. Statistical analyses were performed using IBM SPSS Statistics for Windows, version 26.0 (IBM Corp.; Armonk, NY, USA) and SAS for Windows, version 9.4 (SAS Institute Inc.; Cary, NC, USA).

**Table 1:** Treatment and preventive measure codes used in the study

Treatment code and meaning	The general service provider fees	Description
**101**,** 112**,** 111** Complete Examination	**SEK 792**,** EUR 83**,** USD 94** **SEK 772**,** EUR 81**,** USD 91** **SEK 582**,** EUR 61**,** USD 69**	Examination and investigation performed by a dentist or dental hygienist on one occasion.
**161** Saliva secretion measurement	**SEK 542**,** EUR 57**,** USD 64**	The measure includes information about the sampling, collection of saliva, measurement of secretion values, documentation, and possible consultation responses.
**162** Microbiological examinations	**SEK 302**,** EUR 32**,** USD 36**	The measure only includes laboratory costs for microbiological examinations.
**Disease preventive measures**		
**201** Information or instruction on risk of oral health-related illnesses or problems	**SEK 420**,** EUR 44**,** USD 50**	The measure includes informing the patient about causation to prevent caries or giving detailed instruction regarding self-care.
**204** Prophylaxis in individual trays, per tray	**SEK 730**,** EUR 77**,** USD 87**	The measure includes impression taking as well as the production, testing, and delivery of trays of fluoride gel or chlorhexidine gel home-care treatment.
**205** Fluoride treatment, shorter treatment time	**SEK 155**,** EUR 16**,** USD 18**	The measure includes professional tooth cleaning for biofilm removal, approximal cleaning and required fluoride treatment, and requires approximately 10 min of treatment time.
**206** Fluoride treatment	**SEK 307**,** EUR 32**,** USD 36**	The measure includes professional tooth cleaning for biofilm removal, approximal cleaning and required fluoride treatment, and requires approximately 20 min of treatment time.
**Disease treatment measures**		
**311** Information or instruction for oral health-related illnesses or problems	**SEK 430**,** EUR 45**,** USD 51**	The measure includes information on causation in oral health-related diseases or problems, or detailed instructions regarding self-care.
**312** Follow-up information or instruction in case of oral health-related diseases or problems	**SEK 152**,** EUR 16**,** USD 18**	The measure is eligible for a maximum of six times per patient.
**313** Behavioral change treatment, 60 min or more	**SEK 940**,** EUR 99**,** USD 112**	The measure includes qualified behavioral change approach in managing oral health-related diseases and must contain an individualized treatment plan for theory-based behavioral effects.
**314** Behavioral change treatment	**SEK 455**,** EUR 48**,** USD 54**	The measure includes qualified behavioral change methods in managing oral health-related diseases and must contain an individualized treatment plan for theory-based behavioral effects.
**321** Non-operative treatment of caries disease	**SEK 435**,** EUR 46**,** USD 52**	The measure includes intensive fluoride treatment, antimicrobial treatment, or dietary advice. The measure includes, if applicable, professional tooth cleaning. The measure is eligible for compensation for in-depth dietary advice only if it has its background in a diet anamnesis.

All dental procedures are registered using specific codes. The Dental and Pharmaceutical Benefits Agency determines which treatment codes and fees are eligible for reimbursement. The code is applied on completion of the treatment and/or preventive measure, and a fee is charged. SEK: Swedish krona. Exchange rates for 2015–2016 were approximately SEK/EUR 9.5 and SEK/USD 8.4, based on data from Sveriges Riksbank

## Results

**Fig. 1:**
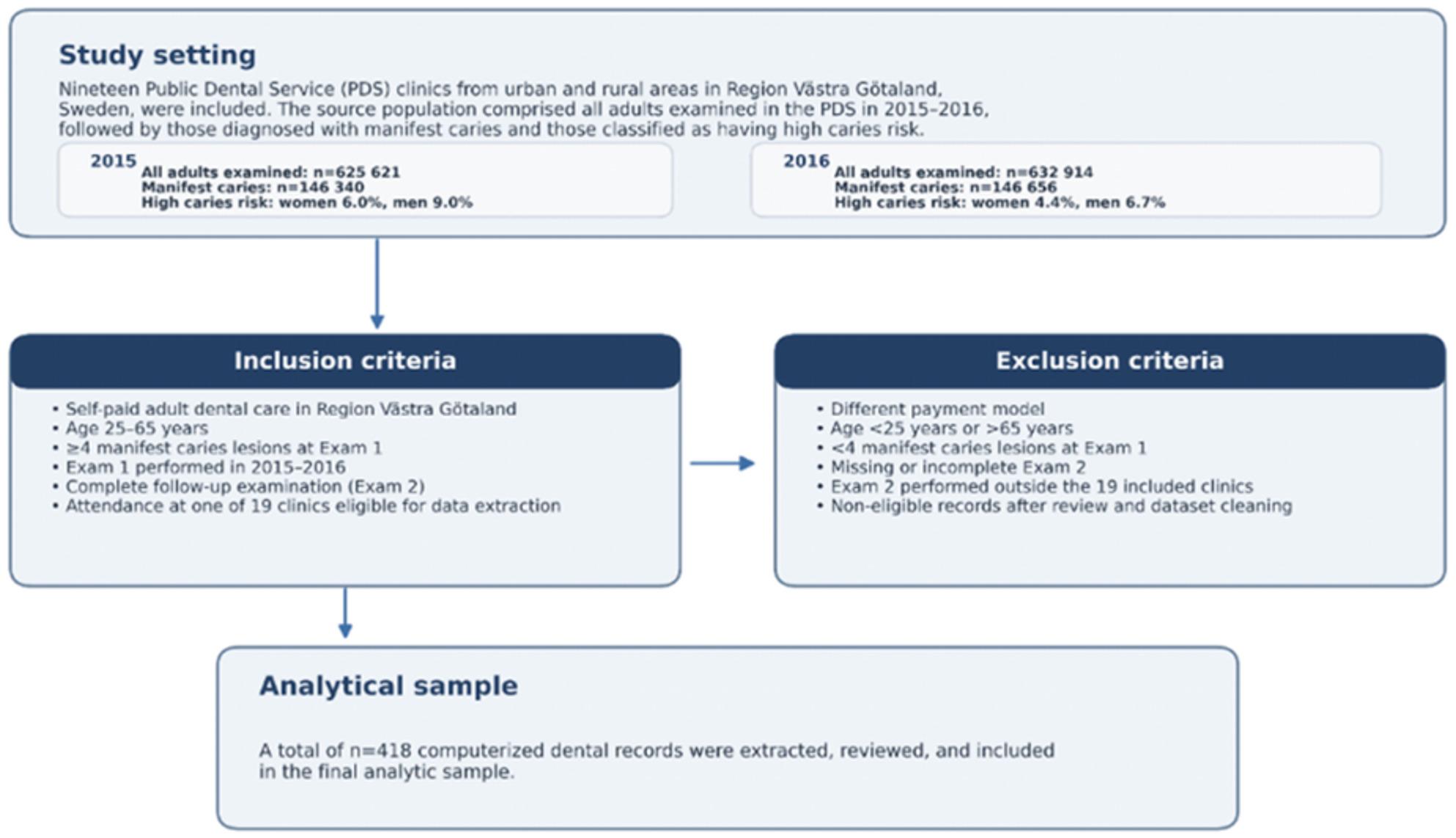
Flow diagram of record selection. Nineteen Public Dental Service clinics in Region Västra Götaland, Sweden, were included. The source population comprised adults examined in the Public Dental Service during 2015–2016. Eligibility criteria were applied to identify adults aged 25–65 years with high caries activity, defined as ≥4 manifest caries lesions at a complete dental examination, who were regular dental attenders and had a subsequent complete examination. After application of inclusion and exclusion criteria, 418 electronic dental records were included in the final analytic sample

The final analytic sample comprised 418 records from highly caries-active adults treated in public dental care in Region Västra Götaland (Fig. 1). The sample included 185 women (44.2%) and 233 men (55.7%), with a mean age of 39 years. Additional baseline characteristics and follow-up intervals are presented in Table 2. Overall, 330 patients (78.9%) had at least one documented preventive and/or treatment measure between Exam 1 and Exam 2, whereas 88 patients (21.1%) had no such documentation. At least one preventive and/or treatment measure was documented in 149 women (80.5%) and 181 men (77.7%). No statistically significant sex-related difference was observed for documentation of any preventive and/or treatment measure (Fisher’s exact test, *p* = 0.546). In the logistic regression model, recorded sex was not significantly associated with documentation of preventive and/or treatment measures after adjustment for age (OR 1.26, 95% CI 0.77–2.03; *p* = 0.352), and no statistically significant sex-by-age interaction was observed (*p* = 0.380). Increasing age was associated with a higher likelihood of documented preventive and/or treatment measures (OR 1.03 per year, 95% CI 1.01–1.06; *p* = 0.010) (Tables 3, 4 and 5).

**Table 2:** General patient characteristics of the study sample by recorded sex and age group (*n* = 418)

Women	Age 25 − 34 *n* = 183 (%)	Age 35 − 44 *n* = 118 (%)	Age 45 − 54 *n* = 71 (%)	Age 55 − 65 *n* = 46 (%)	Total *n* = 418 (%)	Age mean ± sd	Age median (IQR)
	88 (48.0)	52 (44.0)	30 (42.2)	15 (32.6)	185 (44.2)	38 ± 10	35 (30,44)
**Men**	95 (51.9)	66 (55.9)	41 (57.7)	31 (67.3)	233 (55.7)	40 ± 11	37 (31,46)
***p*** **-value**						0.290_A_	0.111_B_

Subscript A: P-value A refers to a chi-square test of homogeneity comparing age-group distribution between women and men. P-value B refers to a Mann–Whitney U-test comparing median age between women and men. Categorical variables are presented as n (%). Continuous variables are presented as mean ± standard deviation (SD) and median (interquartile range, IQR)

**Table 3:** Documented preventive and treatment measures by sex and age

Variable	Women *n* = 185 (%)	Men *n* = 233 (%)	*p*-value	Age 25 − 34 *n* = 183 (%)	Age 35 − 44 *n* = 118 (%)	Age 45 − 54 *n* = 71 (%)	Age 55 − 65 *n* = 46 (%)	*p*-value
**No documented preventive** **and/or** **treatment measures**	36 (19.5)	52 (22.3)	**0.546**	48 (26.2)	24 (20.3)	13 (18.3)	3 (6.5)	**0.030**
**At least one documented preventive and/or treatment measure**	149 (80.5)	181 (77.7)	**0.546**	135 (73.8)	94 (79.7)	58 (81.7)	43 (93.5)	**0.030**
**Number of documented preventive and/or treatment measures**,** mean ± SD**	2.8 ± 2.2	2.8 ± 2.2	**0.930**	2.7 ± 2.4	2.6 ± 2.0	2.9 ± 2.2	3.2 ± 2.1	**0.351**

Subscript *P*-values for differences between women and men were calculated using Fisher’s exact test for dichotomous variables and Mann–Whitney U-test for continuous or ordinal variables.*P*-values for age-group comparisons were calculated using the corresponding tests across age groups

**Table 4:** Documented measures stratified by sex and age group

Variable	Group	Age 25–34 (*n* = 183)	Age 35–44 (*n* = 118)	Age 45–54 (*n* = 71)	Age 55–65 (*n* = 46)	*p*-value
At least one documented preventive and/or treatment measure	Women (total *n* = 149)	67/88 (76.1)	45/52 (86.5)	23/30 (76.7)	14/15 (93.3)	0.451
	Men (total *n* = 181)	68/95 (71.6)	49/66 (74.2)	35/41 (85.4)	29/31 (93.5)	0.020
No documented preventive and/or treatment measures	Women (total *n* = 36)	21/88 (23.9)	7/52 (13.5)	7/30 (23.3)	1/15 (6.7)	0.572
	Men (total *n* = 52)	27/95 (28.4)	17/66 (25.8)	6/41 (14.6)	2/31 (6.5)	0.591
p-values A		0.332	0.049	0.373	0.989	
Number of documented preventive and/or treatment measures, mean ± SD	Women	2.9 ± 2.4	2.7 ± 2.0	2.7 ± 2.0	2.5 ± 1.4	0.403
	Men	2.6 ± 2.3	2.5 ± 2.0	3.1 ± 2.3	3.5 ± 2.3	0.061

Subscript A: Values are presented as n/N (%) unless otherwise stated. *P*-values within sex groups refer to tests for age-related differences. *P*-values A refer to Fisher’s exact test comparing women and men within each age group

**Table 5:** Logistic regression of documented preventive and treatment measures

Dependent variable	Independent variable	Odds ratio 95% Confidence interval	*p*-value
At least one documented preventive and/or treatment measure (Dichotomous with reference category No)	Recorded sex (Dichotomous with reference category Women)	1.26 (0.77–2.03)	0.352
	Age (per increasing year) (Numerical)	1.03 (1.01–1.06)	0.010
***p*** **-values**_**B**_		0.380	

Subscript A: Odds ratios were estimated using binary logistic regression. The dependent variable was documentation of at least one preventive and/or treatment measure. The reference category for recorded sex was women. The interaction between recorded sex and age was tested separately and was not statistically significant (*p*= 0.380)

The distribution of caries-related variables documented in the daily notes is presented in Table 6. In total, 598 preventive measures were documented among the 330 patients with at least one preventive measure recorded between the two examinations. Documentation was concentrated on oral hygiene- and fluoride-related measures, with oral hygiene enquiry, oral hygiene advice, fluoride enquiry, and fluoride advice being the most frequently recorded items. Dietary enquiry and dietary advice were less frequently documented, and documentation of other preventive domains was uncommon, including oral health education related to general health, xerostomia-related measures, and recommendations for high-fluoride toothpaste (Table 6). Of the 171 patients with documented fluoride-related verbal advice, 80 had a documented supplementary self-care recommendation for additional fluoride use at home, indicating that the risk-targeted fluoride recommendations represented a subset of the broader fluoride advice category (Table 6).

**Table 6:** Caries-related documentation extracted from daily clinical notes

Preventive and/or treatment measures obtained from daily notes.	Group *n* = 418 (95% CI)	Women Men *p*-value *n* = 185 (%) *n* = 233 (%)	Age 25–34 Age 35–44 Age 45–54 Age 55–65 *p*-value *n* = 183 (%) *n* = 118 (%) *n* = 71 (%) *n* = 46 (%)
Fluoride treatment at the clinic	65 15.6 (12.2,19.4)	33 (17.8) 32 (13.7) 0.278	32 (17.5) 17 (14.4) 10 (14.1) 6 (13.0) 0.301
Oral hygiene enquiry	265 63.4 (58.6,68.0)	121 (65.4)144 (61.8) 0.481	107 (58.5) 78 (66.1) 46 (64.8) 34 (73.9) 0.150
Oral hygiene, verbal advice	182 43.5 (38.7,48.4)	81 (43.8)101 (43.3) 0.988	82 (44.8) 48 (40.7) 35 (49.3) 17 (37.0) 0.512
Dietary enquiry	144 34.4 (29.9,39.2)	63 (34.1) 81 (34.8) 0.919	63 (34.4) 39 (33.1) 26 (39.6) 16 (34.8) 0.869
Dietary, verbal advice	68 16.3 (12.9,20.2)	24 (13.0) 44 (18.9) 0.110	31 (16.9) 19 (16.1) 12 (16.9) 6 (13.0) 0.565
Fluoride enquiry	132 31.6 (27.1,36.3)	60 (32.4) 72 (30.9) 0.751	53 (29.0) 33 (28.0) 22 (31.0) 24 (52.2) 0.070
Fluoride, verbal advice	171 40.9 (36.2,45.8)	73 (39.5) 98 (42.1) 0.619	75 (41.0) 42 (35.6) 31 (43.7) 23 (50.0) 0.471
Xerostomia enquiry	15 3.6 (2.0,5.8)	4 (2.2) 11 (4.7) 0.192	4 (2.2) 4 (4.4) 5 (7.0) 2 (4.3) 0.161
Xerostomia verbal advice	17 4.1 (2.4,6.4)	7 (3.8) 10 (4.3) 0.991	7 (3.8) 3 (2.5) 4 (5.6) 3 (6.5) 0.852
Information on the association between general and oral health	15 3.6 (2.0,5.8)	7 (3.8) 8 (3.4) 0.990	7 (3.8) 4 (3.4) 3 (4.2) 1 (3.3) 0.813
Behavioral medicine prevention and treatment (behavior change method)	0 0.0 (0.0, 0.9)	0 (0.0) 0 (0.0)	0 (0.0) 0 (0.0) 0 (0.0) 0 (0.0) N/A
Salivary secretion measurement	0 0.0 (0.0, 0.9)	0 (0.0) 0 (0.0)	0 (0.0) 0 (0.0) 0 (0.0) 0 (0.0) N/A
Microbiological test	0 0.0 (0.0, 0.9)	0 (0.0) 0 (0.0)	0 (0.0) 0 (0.0) 0 (0.0) 0 (0.0) N/A
**Supplementary self-care recommendation regarding additional fluoride**	80 19.1 (15.0,22.7)	37 (20.0) 43 (18.5) 0.711	37 (20.2) 18 (15.3) 12 (15.5) 13 (28.3) 0.741
*High-fluoride toothpaste*	23 5.5 (3.3,9.2)	9 (4.9) 14 (6.0) 0.831	11 (6.0) 5 (4.2) 4 (5.6) 3 (6.5) 0.930
*NaF rinse (0*,*2%)*	53 12.7 (9.6,16.3)	25(13.5) 28 (12.0) 0.658	24 (13.1) 12 (10.2) 7 (9.9) 10 (21.7) 0.721
*NaF-CHX gel*	3 0.7 (0.1,2.1)	2 (1.1) 1 (0.4) 0.586	1 (0.5) 1 (0.8) 1 (1.4) 0 (0.0) 0.388
*Fluoride-containing chewing gum*	1 0.2 (0.0,1.3)	1 (0.5) 0 (0.0) 0.437	1 (0.5) 0 (0.0) 0 (0.0) 0 (0.0) 0.849

Subscript A: Values are presented as n (%) or n, % (95% CI). *P*-values for differences between women and men were calculated using Fisher’s exact test. Associations with age were analysed using age as a continuous variable where appropriate. Exact 95% confidence intervals for proportions were calculated using the Clopper–Pearson method. N/A indicates that statistical testing was not applicable because no events were observed

## Discussion

This retrospective review of routine clinical records found limited documented implementation of evidence-based caries treatment and prevention among highly caries-active adults receiving public dental care. Preventive documentation was dominated by oral hygiene enquiry and fluoride advice, whereas guideline-recommended causal and behaviour-oriented strategies were rarely recorded, indicating a gap between national recommendations and documented adult preventive practice [12, 13]. This pattern is consistent with current understanding of dental caries as a behaviourally mediated, non-communicable disease in which effective management requires attention to modifiable drivers of disease and sustained prevention, rather than restorative care alone [2–7, 10, 11]. In the Swedish context, national guidance states that oral health information alone is generally insufficient to change unhealthy habits and recommends advisory counselling or theory-based behaviour change approaches as part of causal caries management [12, 13]. In the present study, no record contained documentation indicating delivery of such approaches, and targeted preventive strategies beyond basic advice were infrequently documented, including diet-focused counselling, in-clinic fluoride regimens, and adjunctive causal investigations. These findings extend previous Swedish research on caries prophylaxis among caries-active adults, which has primarily examined patient-reported outcomes and long-term experiences of preventive care [22]. Recent Swedish qualitative evidence also indicates that experienced dentists may perceive caries prevention for adults with recurrent cavities as difficult to sustain in routine practice [23]. By examining routine clinical records, the present study adds a complementary perspective on whether documented care reflected guideline-aligned causal and behaviour-oriented prevention among highly caries-active adults.

The clinical relevance of the findings lies less in the magnitude of demographic differences and more in the overall documentation pattern among adults with high caries activity. This group would be expected to require causal caries management, including systematic attention to oral hygiene, fluoride exposure, diet, salivary factors, and behaviour-oriented support. Sparse documentation of intensified fluoride measures, dietary counselling, salivary investigation, and theory-based behaviour change approaches may affect continuity of care, accountability, and the ability to evaluate whether guideline-recommended prevention is implemented in routine adult dental practice. In relation to the exploratory null hypotheses, the null hypothesis of no sex-related difference in documentation of preventive and/or treatment measures was not rejected. By contrast, the null hypothesis of no age-related association was rejected, as increasing age was associated with a higher likelihood of documented preventive and/or treatment measures. These analyses should be interpreted as exploratory. The sample-size calculation was designed to provide precision for descriptive estimates of documentation patterns, not to power subgroup comparisons or establish equivalence between women and men.

An important pattern in the findings was the discrepancy between enquiry and active intervention. Dietary habits were more often documented as enquired about than as targets of advice aimed at behaviour change, indicating that risk identification did not consistently translate into documented preventive action. A similar pattern was observed for fluoride-based prevention, in which general advice was common but escalation to higher-intensity measures was uncommon. Taken together, these findings indicate that documented preventive care was often limited to identifying risk factors rather than systematically addressing them.

These findings should be interpreted as reflecting documented rather than verified delivered care. Record-based studies depend on the completeness and consistency of clinical documentation, and under-documentation of non-operative preventive interventions, particularly counselling content and behavioural support, cannot be excluded. This concern is supported by prior Swedish record-review evidence showing frequent absence of key preventive information in dental records [19]. However, documentation is also a component of quality of care because it supports continuity, accountability, and adherence to evidence-based practice. Accordingly, limited documentation of guideline-recommended strategies remains relevant whether it reflects incomplete recording, incomplete delivery, or both [12, 13]. Several mechanisms may plausibly contribute to limited documentation of preventive care in routine adult dental practice, including time constraints, organisational routines, financial incentives, uncertainty about preventive coding, and clinicians’ perceived capability or confidence in delivering preventive counselling [24–26]. These factors were not examined directly in the present study and should therefore be interpreted as hypotheses for future implementation research. An age-related pattern was observed, with older adults more likely than younger adults to have documented preventive measures, while no association was found with sex. The reasons for this pattern cannot be determined from the available data, but several hypotheses are plausible. Older adults may have been perceived as more clinically vulnerable, may have had more complex disease histories or comorbidity, or may have had visit structures that created more opportunities for preventive discussion and documentation. Differences in clinician prioritisation or clinician–patient interaction may also have contributed. These hypotheses should be examined in future studies using visit-level and clinician-level data, including information on visit length, type of appointment, disease severity, and provider characteristics. Further research should also assess whether preventive strategies are delivered and documented equitably across adult age groups. At the system level, the findings are consistent with Swedish reports and guidelines indicating that preventive interventions and causal management remain underused or insufficiently implemented among adults receiving restorative care [12–14]. Future research should evaluate implementation approaches that support more consistent delivery and documentation of targeted, patient-centred preventive care in routine practice [13, 24–26].

### Strengths and limitations

The study has several strengths. It used routine electronic dental records from multiple public dental clinics, included adults with a clearly defined high caries activity threshold, and combined procedure-code data with review of daily clinical notes. The documentation domains were linked to guideline-relevant components of caries management, allowing assessment of whether preventive care was made visible in routine records. This provides a practice-based perspective that complements studies based on patient reports, surveys, or controlled intervention settings.

This study has limitations that should be considered when interpreting the findings. First, the retrospective design relied on routinely collected clinical records, which reflect documented rather than necessarily delivered care. Incomplete or inconsistent documentation may therefore have resulted in underestimation of preventive measures, particularly non-operative and behavioural interventions that are less consistently recorded in clinical notes. The record review was conducted by a single extractor. Although extracted treatment codes were cross-checked against the corresponding clinical notes to enhance validity, no duplicate extraction or formal inter-rater reliability assessment was performed. Some degree of interpretation variability or misclassification therefore cannot be excluded. In addition, some variation in clinical documentation practices may have introduced information bias. Although the study was conducted within one regional public dental organisation with a shared governance context, some variation in how clinicians recorded preventive care and related variables cannot be excluded. Such variation may have affected the completeness and consistency of the recorded information. Detailed information on dentifrice brand and exact fluoride concentration was not consistently available in the records. Interpretation of fluoride-related preventive measures was therefore limited to the documented categories of advice, treatment, and supplementary fluoride recommendations.

Second, the study was conducted within the Public Dental Service in a large but single Swedish region, which may limit generalisability to other healthcare systems, funding models, or organisational contexts. However, the included clinics represent routine public dental care delivered across multiple sites, supporting the relevance of the findings for similar publicly funded dental services. In addition, the study cohort was derived from clinics eligible for data extraction and from patients meeting predefined inclusion criteria within routinely collected records. This may have introduced selection bias if included clinics or included patients differed systematically from other public dental service settings or other highly caries-active adults in the region.

Third, the observational design precludes causal inference regarding relationships between patient characteristics and documented preventive care. The study aimed to describe patterns of documented care and guideline adherence rather than to evaluate the effectiveness of specific interventions.The sample-size calculation was designed to estimate documentation patterns with acceptable precision in the overall sample, rather than to power subgroup analyses by sex or age. Accordingly, the absence of statistically significant sex-related differences should not be interpreted as evidence of equivalence between women and men. The sex distribution was moderately imbalanced, with more men than women included, which may have reduced precision in sex-specific comparisons.

Finally, the data were collected during 2015–2016. Although national guidelines for caries prevention were already established at that time, changes in clinical practice may have occurred since then. The findings should therefore be interpreted as a description of practice during the study period, providing a baseline for evaluating subsequent implementation efforts.

## Conclusions

In this retrospective review of routine public dental records, adults with high caries activity had uneven documentation of caries treatment and preventive measures. Documentation was most evident for oral hygiene- and fluoride-related advice, whereas documentation of causal, intensified, and behaviour-oriented management domains was limited. These findings indicate that routine records only partly reflected guideline-relevant preventive caries management and provide a baseline for future evaluation of documentation and implementation of adult caries prevention in public dental care.

## Supplementary Information

Supplementary Material 1.

## Data Availability

The datasets used and analysed during the current study are available from the corresponding author on reasonable request, subject to regional data protection regulations.

## Abbreviations

CI: Confidence interval
IQR: Interquartile range
OR: Odds ratio
RECORD: REporting of studies Conducted using Observational Routinely collected health Data
SD: Standard deviation
STROBE: Strengthening the Reporting of Observational Studies in Epidemiology
